# Emergence of ST11 *Klebsiella pneumoniae* co-carrying *bla*_KPC-2_ and *bla*_IMP-8_ on conjugative plasmids

**DOI:** 10.1128/spectrum.03345-24

**Published:** 2025-10-08

**Authors:** Lu Gong, Huishuang Yang, Xinrui Wang, Kun Wang, Bingyou Yin, Xiaolu Yang, Haowei Ye, Zhenghao Lou, Tongxi Hu, Weidong Zhu, Beiwen Zheng

**Affiliations:** 1 School of Basic Medical Sciences, Zhejiang Chinese Medical University 659404, Hangzhou, China; 2 Collaborative Innovation Center for Diagnosis and Treatment of Infectious Diseases, State Key Laboratory for Diagnosis and Treatment of Infectious Diseases, the First Affiliated Hospital, Zhejiang University School of Medicine 26441 , Hangzhou, China; 3 The Third Clinical Medical College of Zhejiang Chinese Medicine University, Hangzhou, China; 4 Department of Emergency Medicine, Traditional Chinese Medical Hospital of Zhuji, Zhuji, China; 5 College of Veterinary Medicine, China Agricultural University 630101 , Beijing, China; 6 The First Affiliated Hospital and College of Clinical Medicine of Henan University of Science and Technology, Luoyang, China; 7 Zhejiang Chinese Medical University School of Public Health 668733, Hangzhou, China; 8 Jinan Microecological Biomedicine, Shandong Laboratory 661980 , Jinan, China; 9 Yuhang Institute for Collaborative Innovation and Translational Research in Life Sciences and Technology, Hanghzou, China; JMI Laboratories, North Liberty, lowa, USA

**Keywords:** carbapenem resistance, *Klebsiella pneumoniae*, *bla*_KPC-2_, *bla*_IMP-8_, conjugative plasmid

## Abstract

*Klebsiella pneumoniae* is a major pathogen with substantial antimicrobial resistance driven by β-lactamase production. The co-existence of carbapenemase genes *bla*_KPC-2_ and *bla*_IMP-8_ in the prevalent *K. pneumoniae* clone is rare and poses significant clinical challenges in China. In this study, we report the first identification of a clinical ST11 *K. pneumoniae* strain Kp4874, isolated from a hospitalized patient in China, co-carrying *bla*_KPC-2_ on a ~134 kb IncFII/IncR hybrid plasmid and *bla*_IMP-8_ on a ~75 kb untypable plasmid. Whole-genome sequencing revealed key insertion sequences, including Tn*As1* and IS*26*, facilitating horizontal transfer of these resistance genes. Conjugation experiments confirmed the high transferability of both plasmids, particularly the *bla*_KPC-2_ plasmid. Despite harboring multiple virulence genes, the strain’s clinical threat stems primarily from its multidrug-resistant profile. This study highlights the potential for rapid dissemination of such strains in healthcare settings and underscores the critical need for robust surveillance and infection control measures.

**IMPORTANCE:**

This study is the first to report the co-existence of *bla*_KPC-2_ and *bla*_IMP-8_ in an ST11 *Klebsiella pneumoniae* strain, underscoring the clinical threat posed by these carbapenemase genes. The identification of *bla*_KPC-2_ on an IncFII/IncR hybrid plasmid, coupled with the successful conjugation of both resistance genes, highlights the significant potential for horizontal gene transfer and multidrug-resistant dissemination. These findings advance our understanding of plasmid-mediated resistance and emphasize the urgent need for enhanced monitoring and infection control strategies to mitigate the spread of such high-risk strains.

## INTRODUCTION

Carbapenem-resistant *Klebsiella pneumoniae* (CRKP) has emerged as a significant public health threat due to its extensive antimicrobial resistance and association with high morbidity and mortality, especially in hospital environments. Molecular epidemiological studies have identified clonal complex ST11 as the dominant lineage of CRKP in China, particularly in hospital settings. The clinical strain Kp4874 analyzed here, isolated from a fecal sample of a 69-year-old male patient at a tertiary hospital in Hangzhou, exemplifies the adaptability of ST11 clones to hospital environments and their propensity for acquiring multidrug resistance (1, 2). ST11 is considered a high-risk clone due to its strong association with carbapenem resistance and its capacity for rapid dissemination in healthcare settings. This clonal group is a single-locus variant of the pandemic clone ST258, which is prevalent in North America and Europe (3). However, unlike ST258, which primarily harbors the *bla*_KPC_ gene, ST11 strains in China are often associated with a diverse array of carbapenemase genes, making them highly adaptable and capable of acquiring additional resistance determinants.

Among the carbapenemases contributing to CRKP, KPC-2 is one of the most frequently encountered enzymes, playing a critical role in the spread of resistance worldwide. The *bla*_KPC-2_ is typically located on IncFII and IncR plasmids, which are widely distributed in *Enterobacteriaceae*, including *K. pneumoniae* and *Escherichia coli* (4). IncFII plasmids are among the most globally prevalent resistance plasmids, often carrying multiple resistance genes, thereby increasing the risk of the spread of multidrug-resistant (MDR) strains (4, 5). IncR plasmids are often co-associated with *bla*_KPC-2_ and *bla*_NDM_ and have been widely identified in clinical isolates from China (6). However, hybrid plasmids that carry *bla*_KPC-2_, particularly those combining IncFII and IncR, have been less frequently studied (7), and the mechanisms underlying their fusion remain poorly understood. This knowledge gap limits our understanding of their role in facilitating the transfer and dissemination of resistance genes.

IMP-8, a metallo-β-lactamase (MBL), is another carbapenemase with a distribution that varies by region (8). IMP-type enzymes are especially prevalent in Japan and Southeast Asia (9). However, IMP-8 remains relatively rare in clinical settings in China. Unlike chromosomally encoded resistance genes, *bla*_IMP-8_ is more often plasmid-mediated, which poses a greater threat due to plasmids’ ability to spread resistance through horizontal gene transfer across bacterial populations (10). Chromosomal localization of *bla*_IMP-8_ generally restricts its dissemination, but when found on plasmids, particularly those with strong transfer capabilities, the potential for *bla*_IMP-8_ to spread increases significantly (11). The research on *bla*_IMP-8_, particularly its genetic environment and transferability, remains limited (12), hindering a comprehensive understanding of its role in MDR dissemination.

While *bla*_KPC-2_ and *bla*_IMP-8_ are individually common in clinical isolates, their co-occurrence in a single bacterial strain is exceedingly rare (13). Previous studies have reported *Enterobacterales* isolates co-producing *bla*_KPC_ and MBLs, including *bla*_IMP_ variants, highlighting the growing threat of multi-carbapenemase-producing strains (14). Faccone et al. (15) described the emergence of *Enterobacterales* strains co-producing *bla*_KPC_ and *bla*_IMP_ during the coronavirus disease 2019 (COVID-19) pandemic, demonstrating the ability of such strains to persist and disseminate in hospital environments. However, most reports have focused on species such as *Enterobacter cloacae* and *Pseudomonas aeruginosa* rather than *K. pneumoniae* (15). In this study, we describe a rare case of a *bla*_KPC-2_ and *bla*_IMP-8_ co-producing *K. pneumoniae* isolate, which, to our knowledge, is the first such report in China. The *bla*_KPC-2_-carrying plasmid was identified as an IncFII/IncR hybrid plasmid. This plasmid structure not only harbors multiple resistance genes but also features insertion sequences such as IS*26*, which enhance plasmid transferability (16). Additionally, the *bla*_IMP-8_ gene was located on an untypable plasmid, and its transfer capabilities remain unclear. While *bla*_IMP-8_ has been frequently associated with IncC plasmids, studies on its conjugative efficiency in *K. pneumoniae* are limited (10, 12, 17). The co-existence of *bla*_KPC_ and *bla*_IMP_ significantly enhances bacterial resistance, making treatment options extremely limited. This is the first study to identify these two carbapenemase genes together in a single *K. pneumoniae* strain (14). This finding not only deepens our understanding of MDR isolates but also underscores the potential threat such strains pose in hospital settings. Through whole-genome sequencing and plasmid analysis, this study reveals the complexity of resistance genes carried by this strain, particularly its robust transferability and broad-spectrum resistance to various antibiotics. Therefore, this research underscores the urgent need for continued surveillance of MDR strains and the implementation of effective infection control measures.

## RESULTS

### Antimicrobial susceptibility of Kp4874

**Fig 1:**
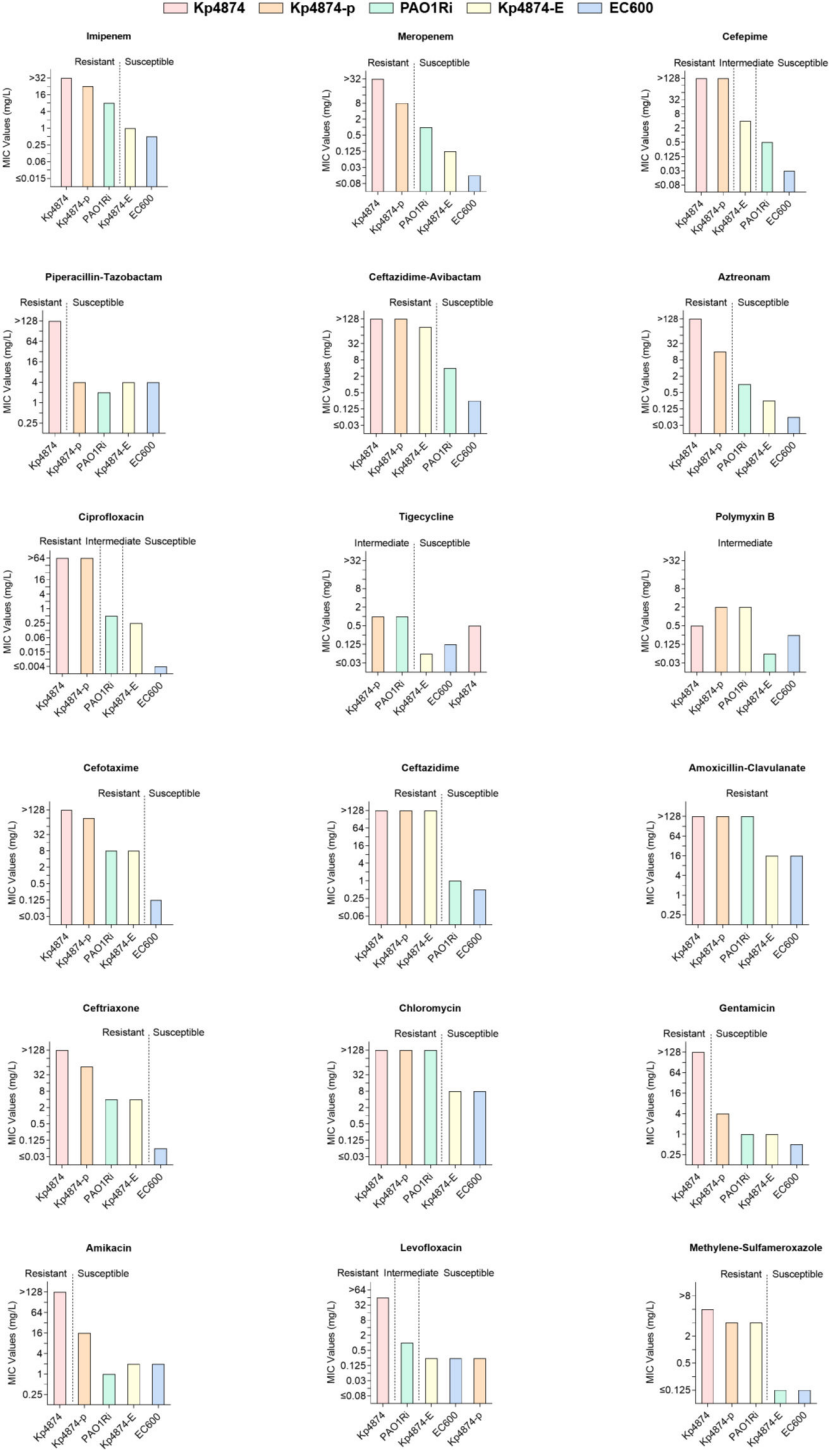
Antimicrobial susceptibilities of strain *K. pneumoniae* Kp4874 and transconjugant minimum inhibitory concentration distributions were separated according to carbapenemase content and are indicated on the histograms. The donor strain is *K. pneumoniae* Kp4874, while the recipient strains are *P. aeruginosa* PAOIRi and *E. coli* EC600. Kp4874-p refers to the conjugant formed between Kp4874 and PAO1Ri, whereas Kp4874-E denotes the conjugant between Kp4874 and EC600.

Antimicrobial susceptibility testing (AST) results showed Kp4874 exhibited extensive multidrug resistance, with high resistance to carbapenems (imipenem, meropenem), cephalosporins (cefotaxime, cefepime, ceftazidime), aminoglycosides (gentamicin, amikacin), and fluoroquinolones (ciprofloxacin, levofloxacin). The presence of resistance genes such as *bla*_KPC-2_ and *bla*_IMP-8_ accounts for the strain’s strong resistance to β-lactam antibiotics. However, Kp4874 remains moderately sensitive to tigecycline and polymyxin B, which may provide potential treatment options (Fig. 1). After conjugation, transconjugant Kp4874-p (*P. aeruginosa* PAO1Ri carrying pKp4874_KPC) acquired high-level resistance to carbapenems and cephalosporins, confirming the role of pKp4874_KPC in mediating resistance. Similarly, transconjugant Kp4874-E (*E. coli* EC600 carrying pKp4874_IMP) displayed increased resistance to imipenem and meropenem compared to the recipient strain, consistent with the presence of *bla*_IMP-8_. The full minimum inhibitory concentration (MIC) values and resistance interpretations are presented in Table S1.

### Plasmid analysis

**Fig 2:**
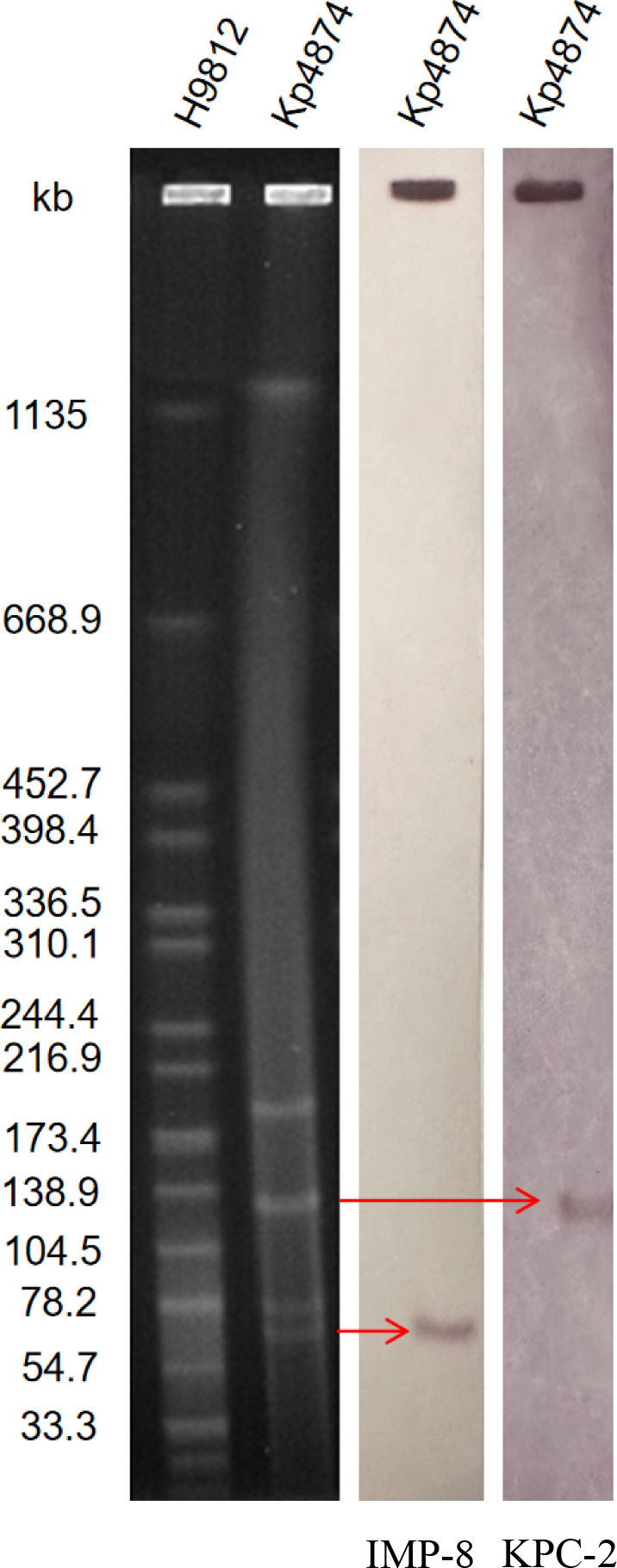
Plasmid profiles and Southern blot hybridization of *K. pneumoniae* strain Kp4874. Southern blot hybridization of S1-nuclease digested DNA using a specific probe (*bla*KPC, *bla*IMP). M: Xbal digested total DNA of *Salmonella enterica* serotype Braenderup H9812 as a size marker.

**Fig 3:**
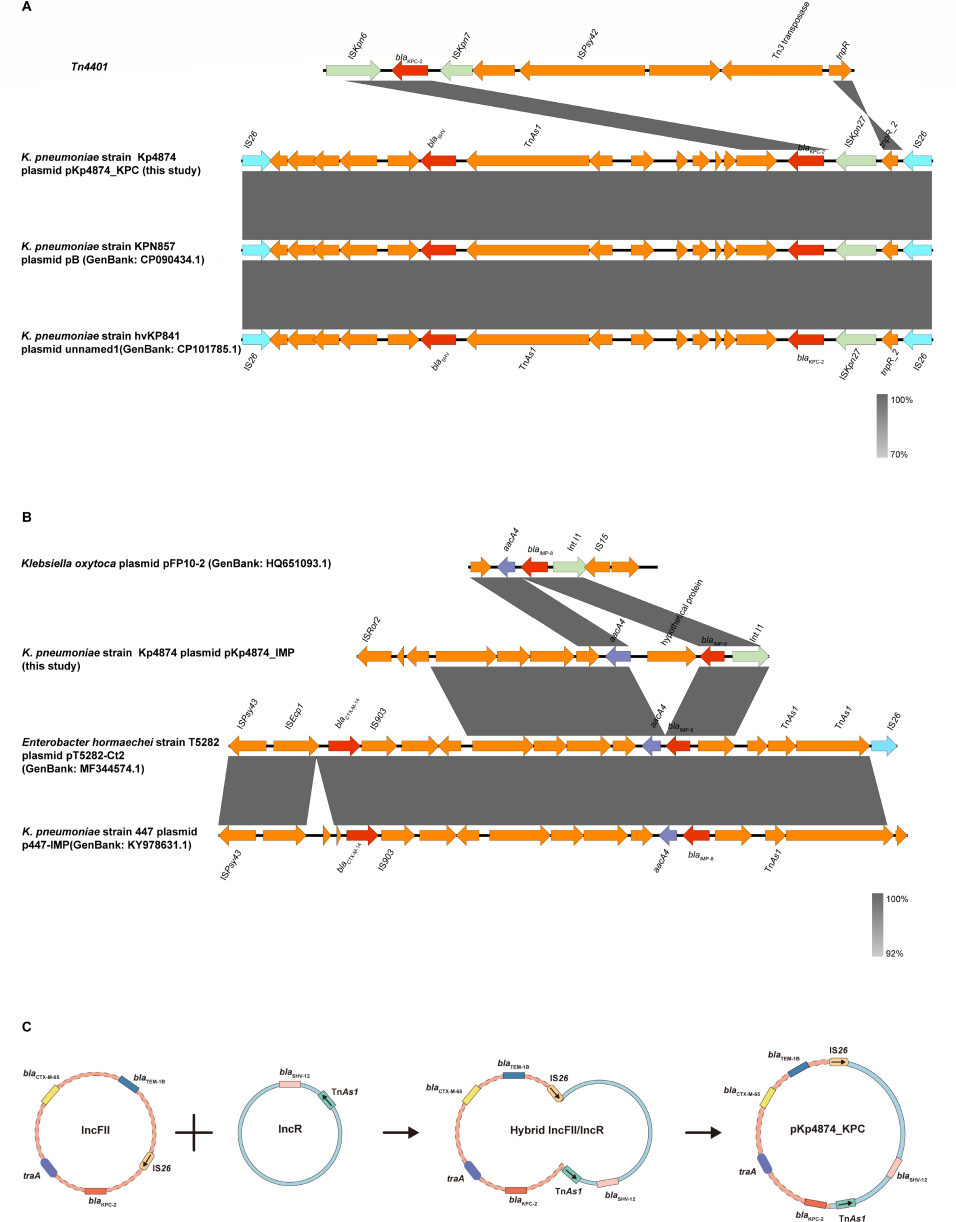
Genetic context of *bla*_KPC-2_ (**A**) and *bla*_IMP-8_ (**B**) on Kp4874 and the formation process of the pKp4874_KPC plasmid (**C**). Arrows denote genes. Genes, mobile elements, and other features are colored based on their functional classification. Panel C illustrates the IS*26*-mediated cointegrate formation process of the hybrid plasmid pKp4874_KPC. Copy-in transposition: IS*26* initiates replicative transposition at a target site between the IncFII and IncR plasmids, generating a cointegrate structure with duplicated IS*26* copies (left to middle panel). Targeted conservative recombination: the transposase Tn*As1* catalyzes site-specific recombination between homologous IS*26* arms, resolving the cointegrate into a stable hybrid plasmid (middle to right panel). This process retains *bla*_KPC-2_ and adjacent resistance genes within a conserved genetic context.

Plasmid analysis via S1-PFGE revealed that the *bla*_KPC-2_ gene resides on a ~134 kb plasmid, while the *bla*_IMP-8_ gene is located on a ~75 kb plasmid (Fig. 2). Plasmid incompatibility groups (IncFII/IncR for *bla*_KPC-2_ and untypable for *bla*_IMP-8_) were determined through whole-genome sequencing and analysis using the PlasmidFinder database. Conjugation experiments demonstrated that both plasmids can be transferred to *P. aeruginosa* and *E. coli*. Successful transfer of both plasmids was confirmed by PCR amplification of *bla*_KPC-2_ and *bla*_IMP-8_ in transconjugants (Fig. S2). While conjugation frequency was not quantified in this study, the consistent recovery of transconjugants across multiple replicates (*n* = 3) supports the functional transferability of both plasmids, particularly the *bla*_KPC-2_-carrying plasmid. The *bla*_KPC-2_ plasmid exhibited robust transferability, as evidenced by the consistent isolation of transconjugants, whereas the *bla*_IMP-8_ plasmid showed lower efficiency. This observation aligns with previous reports highlighting the high conjugation potential of IncFII/IncR plasmids compared to untypable plasmids (6, 18, 19). Whole-genome sequencing classified Kp4874 as sequence type ST11, comprising one chromosome and eight plasmids (Table S1). Genome-based analysis revealed that Kp4874 belongs to the K64 capsule type (KL64 locus) and the O2a O-antigen type (O1/O2v1 locus), with a very high confidence level (99.90% identity). The K64 type is frequently associated with ST11 strains of CRKP, a predominant high-risk clone in China. The O2a serotype has been reported to contribute to immune evasion and persistence in host environments (20, 21). The *bla*_KPC-2_ plasmid was identified as an IncFII/IncR type. Several virulence genes were identified in Kp4874, such as those involved in the type VI secretion system (*clpV*, *icmF*), yersiniabactin biosynthesis (*irp1*, *irp2*), type I and III fimbriae (*fimD*, *mrkC*), and enterobactin synthesis (*entF*), which contribute to its potential virulence. However, genetic analysis alone does not fully determine the strain’s pathogenicity. NCBI BLAST analysis (Fig. S1) showed that the full sequence of plasmid pKp4874_KPC had the highest genetic similarity to *K. pneumoniae* plasmids pKPC84_130120 (CP071162.1, Chengdu, China, clinical), pB (CP090434.1, Guangzhou, China, clinical), unnamed5 (CP101775.1, Zhanjiang, China, clinical), and unnamed1 (CP101785.1, Zhanjiang, China, clinical) (query coverage and homology were both greater than 99%). In contrast, the full sequence of pKp4874_IMP exhibited high genetic similarity to plasmids pF217-3 (CP136728.1, Hangzhou, China, clinical), p2075-2 (CP119167.1, Henan, China, clinical), p2-N3041884 (CP165873.1, São Paulo, Brazil, clinical), and pCfr_tK-N (CP119168.1, Henan, China, clinical) (query coverage and homology were more than 80%). While whole-plasmid BLAST analysis (Fig. S1) identified plasmids with broad genomic homology, Fig. 3 specifically compares the *bla*_IMP-8_ genetic context to plasmids sharing IS*26*-mediated integron structures.

Figure 3 illustrates the gene structures of plasmids pKp4874_KPC (Fig. 3A) and pKp4874_IMP (Fig. 3B), highlighting their respective resistance determinants and mobile genetic elements. These resistance genes are embedded within dynamic genetic regions flanked by IS*26* and Tn*As1*. IS*26* drives plasmid recombination through replicative transposition, forming multidrug resistance clusters (22), as observed in clinical *K. pneumoniae* and *E. coli* isolates. Specifically, IS*26* utilizes “copy-in” and “targeted conservative” mechanisms to mobilize resistance genes (23), exemplified by its role in capturing and disseminating *bla*_CTX-M_ and *bla*_NDM_ genes. The hybrid IncFII/IncR plasmid structure observed here aligns with evolutionary analyses of similar plasmids in *Enterobacteriaceae*, which dominate global MDR outbreaks (18). Notably, IS*26* enhances plasmid transferability by linking multiple resistance genes through transposition or recombination mechanisms, promoting the accumulation and spread of multidrug resistance determinants (24).

The *bla*_KPC-2_ gene is embedded within the Tn*4401* transposon, a well-known mobile genetic element associated with the dissemination of KPC carbapenemases. It is flanked by insertion sequences such as IS*Kpn27*, IS*Kpn6*, and *tnpR*, which further contribute to its mobility. This structural arrangement is consistent with findings that indicate Tn*4401* serves as a significant vehicle for the acquisition and dissemination of *bla*_KPC_-like genes across different bacterial populations (25). Overall, this indicates a robust mechanism for horizontal gene transfer among various *Enterobacteriaceae* species, emphasizing the clinical relevance of monitoring these resistance determinants. The formation of the pKp4874_KPC plasmid is a striking example of such genetic mobility (Fig. 3C). This plasmid arose through the fusion of IncFII and IncR plasmids, mediated by the insertion sequence IS*26* and the transposase Tn*As1*, which facilitated the rearrangement and stabilization of *bla*_KPC-2_ and associated resistance genes. The resulting hybrid IncFII/IncR plasmid highlights the dynamic mechanisms of plasmid recombination and the dissemination of resistance genes. Additionally, integrons, particularly intI1, play a crucial role in the mobility of resistance genes, including *bla*_IMP-8_ (12). The presence of integrons in the genetic background of plasmid pKp4874_IMP suggests that these elements contribute to the acquisition and dissemination of various resistance genes.

### Time-kill analysis and pathogenicity assessment

**Fig 4:**
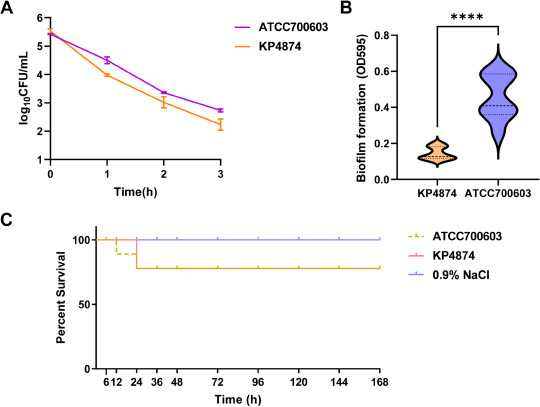
Time-kill curves and pathogenicity characterization of clinical *K. pneumoniae* strains. (**A**) Serum resistance assay of *K. pneumoniae* Kp4874 strain. (**B**) Biofilm formation of *K. pneumoniae* Kp4874 strain. (**C**) Pathogenicity characterization of Kp4874 and ATCC 700603 in the *Galleria mellonella* infection model. **** denotes a statistically significant difference with *P* < 0.0001 compared to the control group.

In the serum resistance assay (Fig. 4A), Kp4874 was classified as level 1 sensitive, indicating a weak ability to evade the host immune response. After 180 minutes of incubation, viable bacterial counts were significantly reduced, suggesting that Kp4874 has difficulty surviving in serum. As shown in Fig. 4B, Kp4874 exhibited significantly lower biofilm formation compared to ATCC 700603, with the difference being statistically significant (*P* < 0.0001). This weak capacity for biofilm formation may limit its ability to establish persistent infections.

In the *Galleria mellonella* infection model (Fig. 4C), larvae injected with Kp4874 had a survival rate comparable to those injected with the control strain ATCC 700603 after 24 hours, with only two larvae succumbing to infection. This finding indicates that, despite Kp4874’s extensive antimicrobial resistance, its pathogenicity in this model is not significantly elevated compared to the non-resistant control strain. The similarity in survival rates suggests that while Kp4874’s multidrug resistance mechanisms allow it to withstand various antibiotics, these traits do not necessarily translate into increased virulence or infectivity *in vivo*. This observation aligns with the results from biofilm formation and serum resistance assays, reinforcing the notion that Kp4874’s clinical significance may stem more from its resistance profile than from an enhanced ability to cause severe infections.

## DISCUSSION

This study highlights the clinical significance of *K. pneumoniae* strain Kp4874, an extensively MDR pathogen carrying both *bla*_KPC-2_ and *bla*_IMP-8_ carbapenemase genes. Both *bla*_KPC-2_ and *bla*_IMP-8_ were successfully conjugated, indicating their potential for horizontal gene transfer, but their transfer characteristics may still differ due to potential structural differences between the plasmids carrying these genes. *Bla*_IMP-8_ is typically located on highly transferable plasmids, often associated with insertion sequences like IS*26* and IS*Kpn27*, which facilitate horizontal gene transfer. In contrast, *bla*_KPC-2_ resides on an IncFII/IncR plasmid, which is known for its robust transfer capabilities and its association with multiple resistance genes (6).

The genetic structure surrounding *bla*_KPC-2_ in pKp4874_KPC exhibits characteristics typical of both IncFII and IncR plasmid types. IncFII plasmids are recognized for their capacity to carry multiple resistance genes and mobile elements that facilitate horizontal gene transfer, thus promoting the spread of resistance within bacterial populations. On the other hand, IncR plasmids generally lack independent conjugative machinery and rely on other plasmids for transfer, typically containing essential resistance genes in a streamlined structure without additional conjugation-related components. This complementary arrangement between IncFII and IncR plasmids enhances the co-transfer of resistance genes, significantly increasing the potential for multidrug resistance. Previous studies have demonstrated that IncFII plasmids exhibit high conjugation efficiency and play a major role in disseminating carbapenemase genes among *Enterobacteriaceae* (8, 26). Notably, Fu et al. (4) reported that IncFII-type plasmids carrying *bla*_KPC-2_ were responsible for the rapid spread of ST11 CRKP in China. Similarly, other studies have shown that hybrid IncFII/IncR plasmids are highly transmissible and frequently associated with multidrug-resistant *K. pneumoniae* strains (13, 27–29). The conjugation experiments conducted in this study support these observations, demonstrating the robust transferability of the *bla*_KPC-2_-carrying plasmid. Our findings align with previous studies demonstrating the role of IncFII/IncR hybrid plasmids in the dissemination of carbapenem resistance genes. Several studies have reported *bla*_KPC-2_-harboring IncFII/IncR plasmids across multiple species, including *K. pneumoniae*, *E. coli*, and *E. cloacae*, in various geographic regions (30–35). These plasmids frequently co-harbor additional antimicrobial resistance (AMR) determinants, forming efficient multidrug resistance dissemination platforms in clinical settings. IS*26* and Tn*As1* are key elements driving the structural evolution of IncFII/IncR plasmids. Previous studies have demonstrated that IS*26*-mediated recombination events facilitate the stabilization and dissemination of *bla*_KPC-2_, often leading to the truncation of transposon structures such as Tn*6296* (30–32). Additionally, Tn*As1* and IS*26* have been implicated in the fusion of IncFII/IncR and IncC plasmids, allowing *bla*_KPC-2_ to be mobilized under selective pressure (33). The presence of such genetic elements underscores the dynamic nature of these plasmids in response to antimicrobial selection. Furthermore, a recent *in vitro* gut model demonstrated that an IncFII/IncR plasmid carrying *bla*_KPC-2_ could be transferred from *K. pneumoniae* to *E. coli*, further supporting the hypothesis that these plasmids play a crucial role in the interspecies dissemination of carbapenem resistance (35). The study also observed that under antibiotic pressure, carbapenemase-producing *Enterobacterales* populations increased significantly, indicating that selection pressure contributes to the persistence and dominance of *bla*_KPC-2_-harboring strains. These studies reinforce the role of IS*26* and Tn*As1* in the evolution of IncFII/IncR plasmids, facilitating their recombination, stabilization, and interspecies transfer. The widespread distribution of such plasmids across multiple bacterial species and geographical locations highlights their epidemiological importance in the global spread of carbapenem resistance.

The differential transferability of the two plasmids can be attributed to host range restrictions and plasmid incompatibility. IncFII/IncR plasmids are primarily found in *Enterobacteriaceae* and are not well-adapted to non-*Enterobacteriaceae* hosts such as *P. aeruginosa* (4, 6, 18). Conversely, *bla*_IMP-8_-carrying plasmids are frequently associated with IncC-type plasmids, which have a broad host range, including *Pseudomonas* spp. (17). This could explain why the *bla*_KPC-2_ plasmid was successfully transferred to *E. coli*, whereas the *bla*_IMP-8_ plasmid was preferentially maintained in *P. aeruginosa*. Additionally, plasmid incompatibility could have influenced these outcomes, as *E. coli* may already harbor IncF plasmids, restricting the stable maintenance of additional IncF-type plasmids.

Notably, the plasmid carrying *bla*_IMP-8_ could not be classified, signaling the need for further investigation into unclassified plasmids to better understand their role in resistance transmission. Unlike previous reports suggesting IS*26*-mediated disruptions, in this study, *bla*_IMP-8_ is positioned upstream of the integron intI1, without evidence of direct disruption by IS*26*. Instead, the gene is embedded within a class 1 integron, a structure known for capturing and mobilizing resistance determinants across bacterial populations (36). Based on our Integron Finder analysis (Table S4), we identified an *attC* site upstream of *bla*_IMP-8_, while no *attC* site was detected downstream. This finding suggests that *bla*_IMP-8_ may have once been part of a gene cassette but has undergone genetic rearrangement, leading to the loss of its typical mobile gene cassette structure. The upstream positioning of *bla*_IMP-8_ relative to intI1 is atypical for class 1 integrons, where gene cassettes are usually found downstream of intI1 within the integron platform. This unusual arrangement raises the possibility of past recombination events or structural modifications that led to the integration of *bla*_IMP-8_ into the current genomic context. Previous reports in the INTEGRALL database have documented *bla*_IMP-8_ as part of a gene cassette, often associated with *aacA4* (37). However, in our study, *bla*_IMP-8_ is found in a distinct arrangement, with *aacA4* oriented in the opposite direction. This inversion or structural variation could be the result of past recombination events, leading to a divergence from previously described integron structures. The absence of a downstream *attC* site suggests that *bla*_IMP-8_ may have lost its ability to be mobilized as part of an integron-mediated recombination process. The presence of this integron highlights its role as a genetic platform for the acquisition and transfer of antimicrobial resistance genes. The results revealed that while our plasmid shares partial sequence similarity with IncC plasmids, it lacks key IncC markers, including *repA* (IncC replication initiator), the A/C-2 replication region, and the mob complex (conjugative transfer system). The absence of these markers suggests that this *bla*_IMP-8_-carrying plasmid is not a typical IncC plasmid but may have originated from an IncC-related ancestor through recombination. Previous studies have demonstrated that *bla*_IMP-8_ can integrate into diverse plasmid backbones, including IncC, IncA, and IncN, emphasizing its genetic mobility and adaptability (38). Moreover, the lack of a complete mob complex suggests that this plasmid may have limited conjugation ability, which could influence its horizontal transfer potential. This raises two possibilities: the plasmid may represent an unclassified or recombinant plasmid that has integrated resistance determinants via a class I integron, or it may be a highly divergent IncC plasmid that has undergone significant structural modifications. Further genome alignment and comparative analyses with IncC reference plasmids are necessary to elucidate its origin (17). For example, a study by Pérez-Vazquez et al. (39, 40) reported the presence of IncC plasmids in the *Klebsiella oxytoca* complex in Spain, with similar findings in China regarding *bla*_NDM_-carrying IncC plasmids in the *K. oxytoca* complex. Conjugation experiments confirmed the horizontal transfer ability of these plasmids, which is a critical concern for the spread of carbapenem-resistant bacteria. Additionally, the cgPMLST 3.5 type of IncC plasmids has been shown to have a wide distribution across several countries, indicating that this plasmid type serves as a significant vehicle for the global spread of antimicrobial resistance (37). Notably, *bla*_IMP-69_ emerged in a context of Tn*402* transposon-mediated movement, which highlights the adaptability of these plasmids and their ability to acquire and disseminate resistance genes. Given these findings, we strongly support the idea that future studies should focus on the comparative genomic analysis of IncC plasmids, particularly to explore their potential role in the evolution and transmission of *bla*_IMP_ and other resistance genes. This would help to better understand their origin, evolution, and potential to spread resistance on a global scale. This finding aligns with the broader observation of *bla*_IMP_-type carbapenemases being regionally variable, with *bla*_IMP-8_ remaining relatively rare in China compared to *bla*_KPC-2_. The *bla*_KPC-2_-carrying plasmid, however, is well-documented in clinical outbreaks, especially in China, where it is associated with hospital-acquired infections (29). The global spread of KPC-producing *K. pneumoniae* highlights the increasing challenge of carbapenem resistance in clinical settings. KPC enzymes, particularly KPC-2 and KPC-3, have been associated with hospital outbreaks and MDR infections, with plasmid-mediated transfer playing a crucial role in their dissemination. In particular, ST258 and ST11 have emerged as dominant KPC-producing lineages, with ST258 being prevalent in the USA and Europe, while ST11 is the predominant lineage in China and other parts of Asia (41). The role of mobile genetic elements such as IncFII, IncN, and IncL/M plasmids has been crucial in facilitating the horizontal transfer of *bla*_KPC-2_, enabling its persistence in various *Enterobacteriaceae* species (42). Additionally, studies from Italy and Israel have demonstrated the rapid emergence and establishment of KPC-producing strains in healthcare settings, underlining the importance of active surveillance, infection control measures, and antimicrobial stewardship to mitigate their spread (41, 43). The co-occurrence of *bla*_KPC-2_ and *bla*_IMP-8_ on separate conjugative plasmids not only amplifies the strain’s resistance profile but also poses a significant challenge for clinical treatment and infection control strategies.

The identification of Kp4874 as K64/O2a is significant, as K64 is one of the most prevalent capsule types in ST11 CRKP isolates, which are known for their high transmissibility and multidrug resistance. The K64 capsule has been associated with increased survival in harsh environments, possibly contributing to bacterial persistence in hospital settings. Moreover, the O2a O-antigen type has been linked to immune evasion, which may enhance bacterial fitness in the host. Together, these serotypes may provide Kp4874 with both enhanced survival mechanisms and multidrug resistance, emphasizing the clinical importance of this strain (20, 21). Although Kp4874 harbors multiple virulence factors, its clinical impact appears to be more closely associated with its MDR profile than with increased virulence. Research indicates that MDR strains do not always demonstrate heightened virulence; instead, their clinical significance often arises from their resistance capabilities. The extensive antimicrobial resistance profile of Kp4874, coupled with the absence of hypervirulence markers (*iuc*, *rmpA*) and its nosocomial origin, strongly supports its classification as a classical *K. pneumoniae* (cKP) strain. Classical strains are typically characterized by multidrug resistance and adaptation to hospital environments, contrasting with hypervirulent lineages that prioritize virulence over resistance (21). While hvKP strains rely on aerobactin and other siderophores for iron acquisition in systemic infections, the lack of these determinants in Kp4874 aligns with its role as a hospital-adapted pathogen. This distinction underscores the divergent evolutionary trajectories of cKP and hvKP, with the former posing challenges in clinical management due to limited therapeutic options, and the latter threatening public health through invasive community outbreaks (44, 45). The co-existence of resistance genes, such as *bla*_KPC-2_ and *bla*_IMP-8_, along with the strain’s capacity for biofilm formation, presents a substantial challenge for antibiotic treatment. However, the strain’s moderate serum resistance and the lack of elevated virulence observed in the *G. mellonella* model suggest that the primary threat posed by Kp4874 lies in its treatment resistance rather than its pathogenicity. This distinction carries important clinical implications (46), as the evolution of resistance does not necessarily correlate with increased virulence, which complicates the management of infections caused by such strains (47). Effectively managing infections by such strains may therefore rely on advanced therapeutic strategies, including combination therapies, as the complexity of multiple resistance genes complicates treatment options and underscores the need for new antimicrobial agents and robust infection control measures.

The identification of highly similar plasmids in multiple Chinese cities and in Brazil suggests that these plasmids may be widespread in hospital settings. This aligns with previous reports of KPC-producing and IMP-producing plasmids contributing to nosocomial outbreaks. The presence of closely related plasmids in geographically distant locations further highlights the potential for international dissemination of multidrug-resistant plasmids, emphasizing the need for enhanced surveillance and infection control measures. In conclusion, the co-occurrence of *bla*_KPC-2_ and *bla*_IMP-8_ in *K. pneumoniae* represents an escalating public health threat. The high transmission potential of these genes, particularly on plasmids with efficient transfer mechanisms, raises the risk of widespread hospital outbreaks (27). Stricter infection control policies and continuous surveillance are essential to curbing the spread of such MDR strains. Future research should focus on understanding the transmission dynamics of *bla*_IMP-8_-carrying plasmids and the prevalence of strains co-harboring *bla*_KPC-2_ and *bla*_IMP-8_ in different clinical settings. Investigating plasmid structures and transfer conditions will be crucial for developing effective infection control strategies. Additionally, ongoing surveillance of resistance patterns and novel therapeutic approaches will be vital in combating the rise of MDR *K. pneumoniae*.

## MATERIALS AND METHODS

### Bacterial isolation and identification

An MDR *K. pneumoniae* strain Kp4874 was isolated from the fecal sample of a 69-year-old male patient at a tertiary hospital in Hangzhou, China. The patient was diagnosed with infective endocarditis, characterized by vegetation formation. Empiric antimicrobial therapy was initiated with vancomycin, followed by rifampin and levofloxacin to optimize treatment efficacy. Despite targeted antibiotic therapy, surgical intervention was ultimately required. The patient underwent cardiac surgery and was subsequently discharged. Species identification and carbapenemase-encoding genes detection were performed using matrix-assisted laser desorption/ionization time-of-flight mass spectrometry (MALDI-TOF MS) and polymerase chain reaction (PCR) amplification (11, 12).

### AST

AST was conducted using agar dilution and broth microdilution methods. The results were interpreted according to the Clinical and Laboratory Standards Institute guidelines (48).

### Plasmid analysis

Plasmid number and size were determined by S1-PFGE. The locations of the *bla*_KPC-2_ and *bla*_IMP-8_ genes were identified through Southern blotting using specific dig-labeled probes (49, 50). Conjugation experiments were conducted using rifampicin-resistant *P. aeruginosa* PAO1Ri (51) and *E. coli* EC600 (52) as recipients. Selective media for transconjugants included 200 mg/L rifampicin with 2 mg/L meropenem for *bla*_KPC-2_ and 64 mg/L rifampicin with 10 mg/L ceftazidime for *bla*_IMP-8_. Successful transfer of both plasmids was confirmed by PCR amplification of *bla*_KPC-2_ and *bla*_IMP-8_ in transconjugants. While conjugation frequency was not quantified in this study, the consistent recovery of transconjugants across multiple replicates (*n* = 3) supports the functional transferability of both plasmids, particularly the *bla*_KPC-2_-carrying plasmid. Finally, PCR amplification was performed to confirm the successful transfer of both plasmids to recipient strains. Primers (Table S2) were used under the following conditions: initial denaturation at 95°C for 5 min, followed by 30 cycles of 95°C for 30 s, 43°C/57°C for 45 s, and 72°C for 7 min, with a final extension at 72°C for 30 s (13).

### Whole-genome sequencing

Total DNA was extracted using the OMEGA Bacterial DNA Kit and sequenced on the Illumina NovaSeq 6000 and Oxford Nanopore platforms. Genome assembly was performed using Unicycler v.0.4.2 (53, 54). Antibiotic resistance genes and plasmid incompatibility types were identified through the ResFinder and PlasmidFinder databases. Plasmid incompatibility groups (Inc groups) were identified using the PlasmidFinder tool (https://cge.food.dtu.dk/services/PlasmidFinder/) with a threshold of 95% identity and 60% coverage (55). The IncFII/IncR hybrid structure of the *bla*_KPC-2_ plasmid was further confirmed by alignment with reference plasmids in the NCBI database. MLST (https://cge.food.dtu.dk/services/MLST/) was determined by NCBI BLAST (56) to identify plasmids related to pKp4874_KPC and pKp4874_IMP. The K (capsule) and O (O-antigen) serotypes of Kp4874 were determined using the Kleborate v.3 tool (https://github.com/katholt/Kleborate) based on whole-genome sequencing data. The prediction was performed using the default settings, and results were interpreted according to the Kleborate reference database (57). Plasmid conjugation was validated using OriTFinder (https://bioinfo-mml.sjtu.edu.cn/oriTfinder), virulence factors were analyzed through the VFDB, and genome annotation was done using Proksee. Plasmid maps were generated using BRIG (58), and resistance gene background comparisons were made using Easyfig.

### Pathogenicity and time-kill assay

The pathogenicity of the collected *K. pneumoniae* isolates was evaluated using serum resistance and biofilm formation assays. Biofilm formation was detected by crystal violet staining, with ATCC 700603 used as the negative control. Each experiment was performed in triplicate (59). Following strain revival on lysogeny broth (LB) agar, a single colony was inoculated into 5 mL LB broth and incubated at 37°C with shaking (200 rpm) for 6 h. Bacterial suspensions were adjusted to a 0.5 McFarland standard (OD_600_ = 0.45-0.55), and 150 µL aliquots were dispensed into triplicate wells of a 96-well plate, with ATCC 700603 as the positive control and sterile LB broth as the negative control. After 24 h of static incubation at 37°C, planktonic cells were removed by gentle aspiration, and biofilms were washed three times with 200 µL phosphate-buffered saline (PBS) (pH 7.4). Plates were air-dried, fixed with 200 µL methanol (15 min, room temperature [RT]), and stained with 0.1% (wt/vol) crystal violet (15 min, RT). Unbound dye was removed via three PBS washes, and biofilm-bound crystal violet was solubilized in 100 µL dimethyl sulfoxide (DMSO) for 10 min with gentle shaking. Absorbance was measured at 550/595 nm using a microplate reader, normalized against negative controls (LB broth) (60, 61).

Additionally, *in vitro* virulence was assessed by a serum killing assay as previously described. Briefly, 25 µL of bacterial suspension (1 × 10^6^ CFU/mL) was added to 75 µL of pooled serum from 10 healthy individuals and incubated in a microtiter plate. Viable counts were measured by plating at 0, 1, 2, and 3 hours (62, 63). The test was repeated at least three times, and serum resistance was characterized based on CFU percentages, classified from grade 1 to 6. Strains reaching grades 5–6 in normal human serum were typically considered serum-resistant. A two-tailed unpaired Student’s *t*-test was used for statistical analysis, and data were expressed as mean ± standard deviation.

### *Galleria mellonella* infection model

For the *G. mellonella* infection model, mid-log phase cultures of *K. pneumoniae* Kp4874 were adjusted to 0.5 McFarland standard in PBS and further diluted to a final density of 10^6^ CFU/mL. Each larva (10 per group) was injected with 10 µL of bacterial suspension, and survival rates were recorded every 24 hours (64). All experiments were conducted in triplicate, and Kaplan-Meier survival curves and log-rank tests were used for statistical analysis.

## Data Availability

The GenBank submission IDs are SAMN41811235 and SAMN41811276.

## SUPPLEMENTAL MATERIAL

Fig. S1 spectrum.03345-24-s0001.tif Major structural features and comparison of β-lactamase-encoding plasmids.

Fig. S2 spectrum.03345-24-s0002.tif PCR confirmation of *bla*_IMP-8_ and *bla*_KPC-2_ in transconjugants.

Fig. S3 spectrum.03345-24-s0003.tif PCR validation of IS*26*-mediated cointegrate formation and the presence of Tn*As1*.

Table S1 spectrum.03345-24-s0004.docx Antimicrobial susceptibilities of strain *K. pneumoniae* Kp4874 and transconjugant.

Table S2 spectrum.03345-24-s0005.docx The genomic characteristics of Kp4874.

Table S3 spectrum.03345-24-s0006.docx Amplification primers for IS*26* and Tn*As1***.**

Table S4 spectrum.03345-24-s0007.docx Integron Finder analysis results.

ASM does not own the copyrights to Supplemental Material that may be linked to, or accessed through, an article. The authors have granted ASM a non-exclusive, world-wide license to publish the Supplemental Material files. Please contact the corresponding author directly for reuse.
