# Supplementary figures and images for: Emergence of ST11 Klebsiella pneumoniae co-carrying blaKPC-2 and blaIMP-8 on conjugative plasmids
Source: Microbiol Spectr. 2025 Oct 8;13(11):e03345-24. doi: 10.1128/spectrum.03345-24 (PMC12584672; doi:10.1128/spectrum.03345-24)

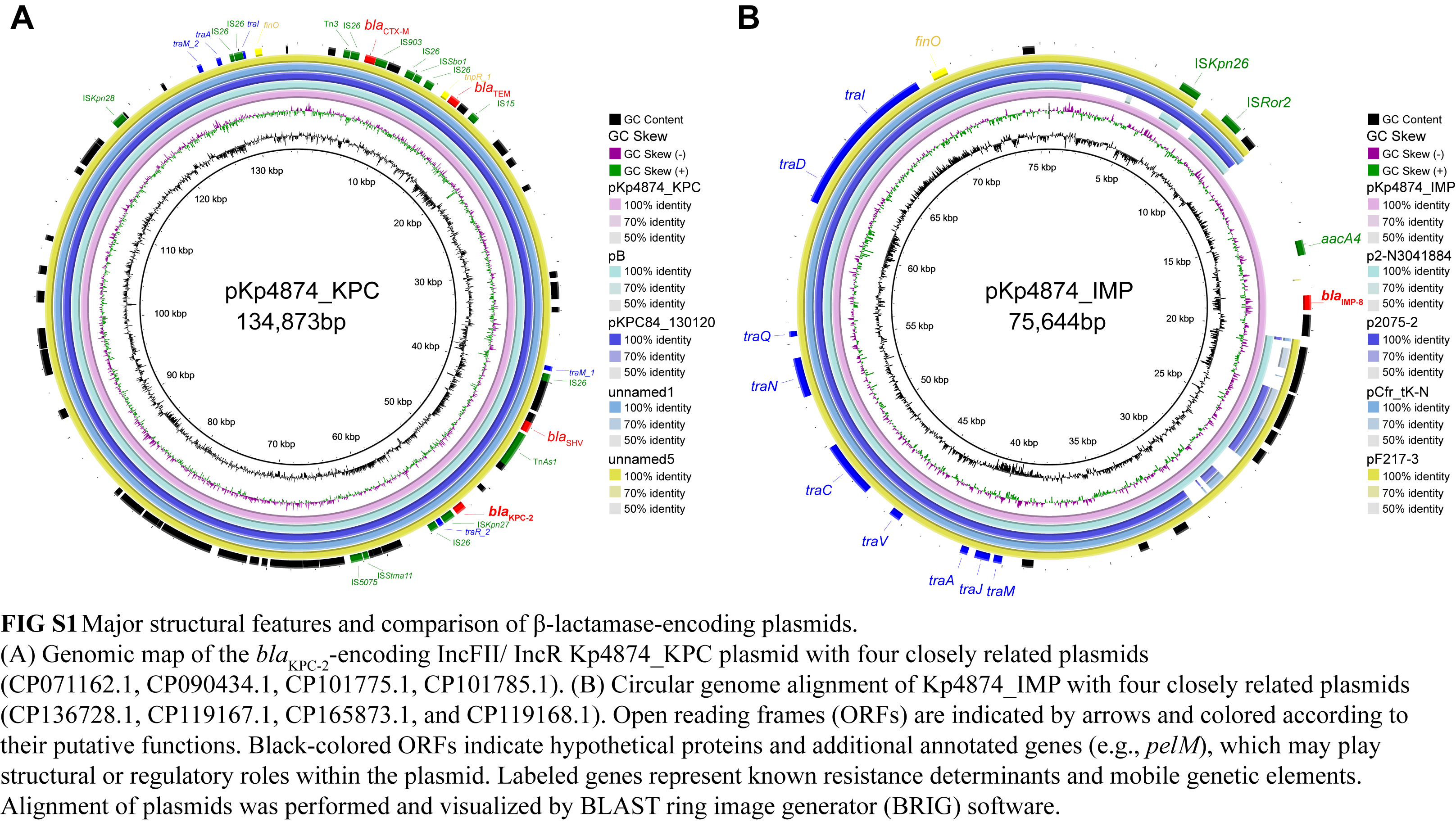

Supplement: Fig. S1 — Major structural features and comparison of β-lactamase-encoding plasmids. [file spectrum.03345-24-s0001.tif]

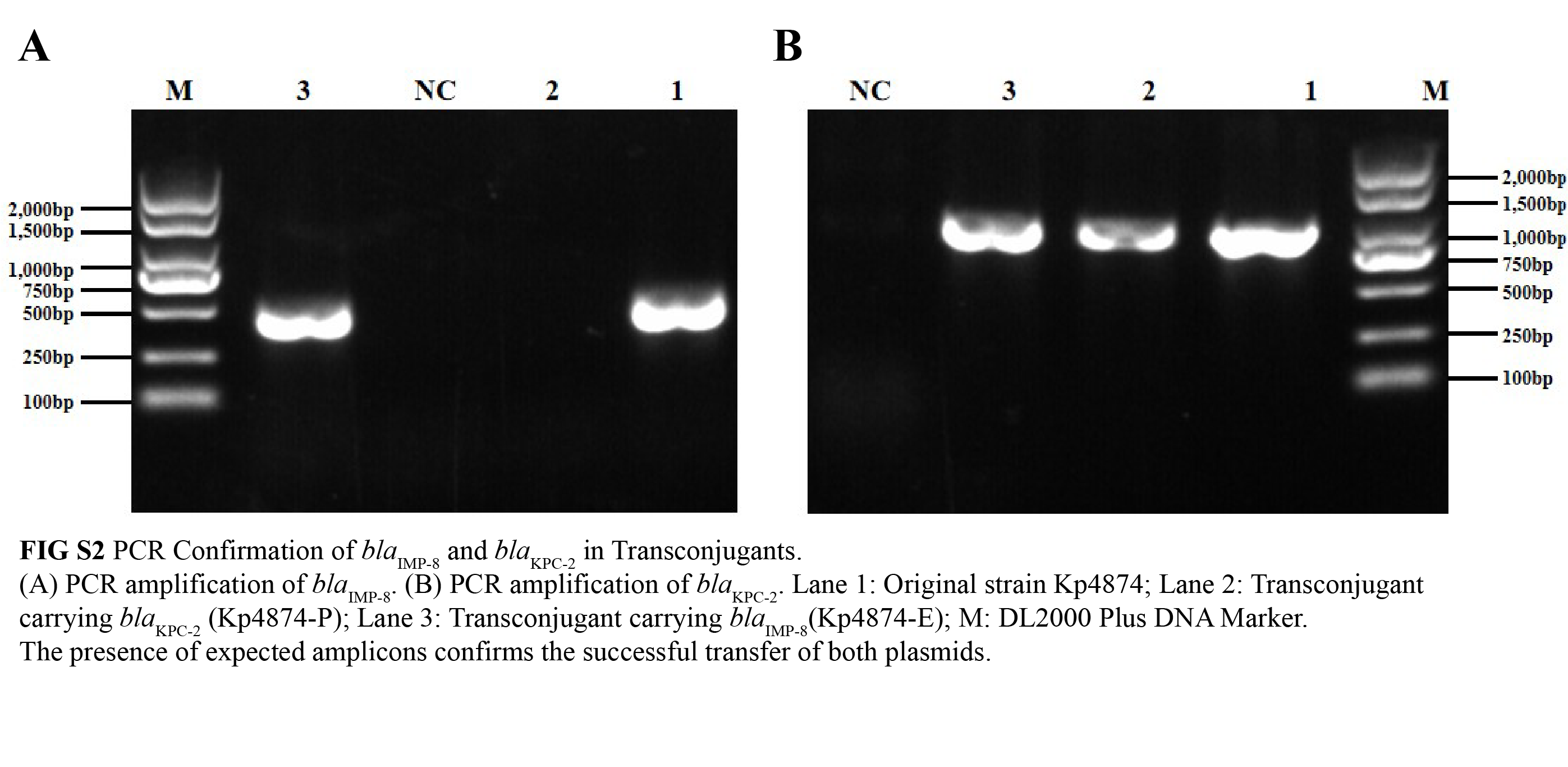

Supplement: Fig. S2 — PCR confirmation of blaIMP-8 and blaKPC-2 in transconjugants. [file spectrum.03345-24-s0002.tif]

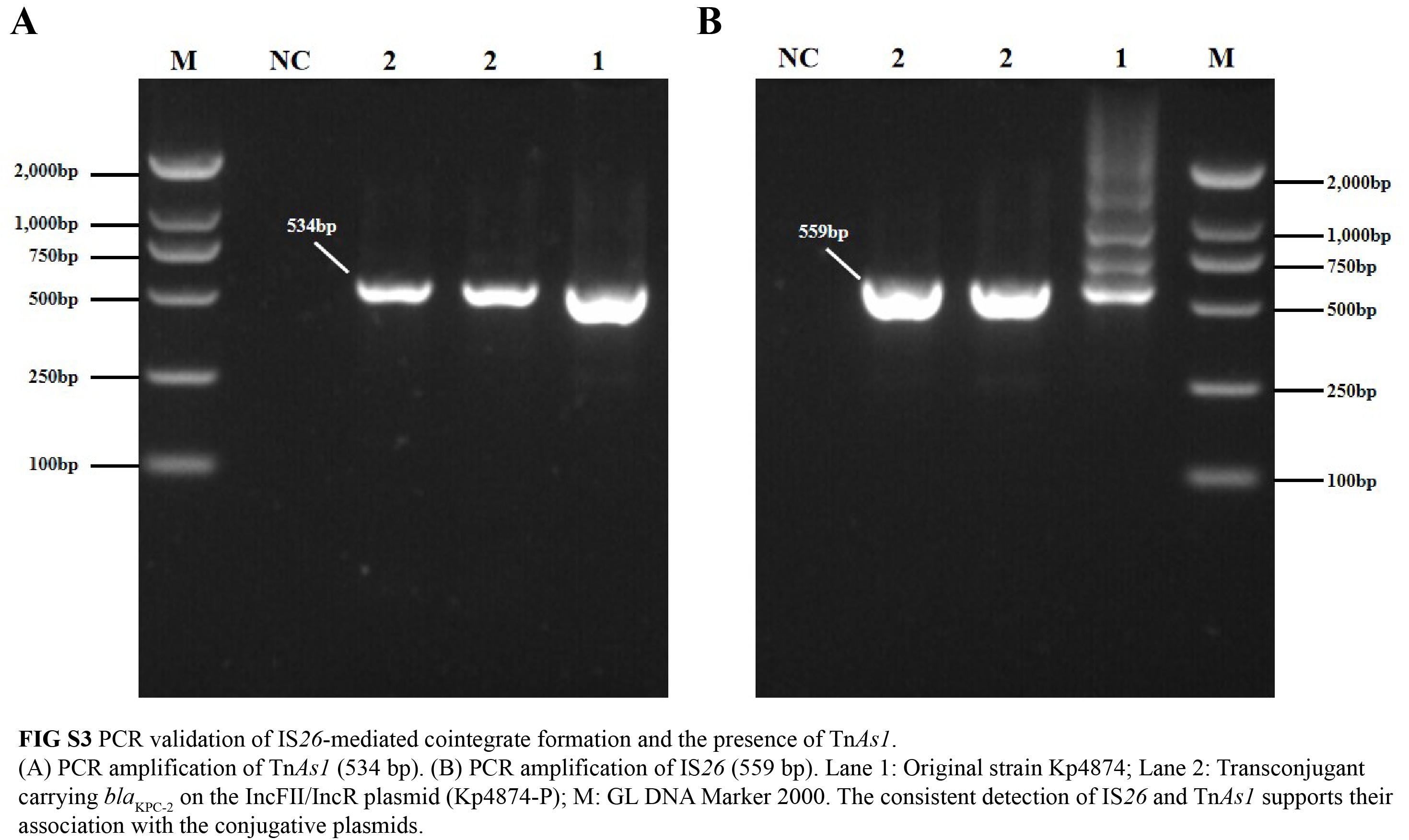

Supplement: Fig. S3 — PCR validation of IS26-mediated cointegrate formation and the presence of TnAs1. [file spectrum.03345-24-s0003.tif]
