# Supplementary material for: Emergence of ST11 Klebsiella pneumoniae co-carrying blaKPC-2 and blaIMP-8 on conjugative plasmids
Source: Microbiol Spectr. 2025 Oct 8;13(11):e03345-24. doi: 10.1128/spectrum.03345-24 (PMC12584672; doi:10.1128/spectrum.03345-24)
Supplement: Table S1 — Antimicrobial susceptibilities of strain K. pneumoniae Kp4874 and transconjugant. [file spectrum.03345-24-s0004.docx]

**Table S1** Antimicrobial Susceptibilities of Strain *K. pneumoniae* Kp4874 and transconjugant

| Antimicrobials | MIC Values (mg/L) | | | | |
| --- | --- | --- | --- | --- | --- |
|  | Kp4874 | Kp4874-p | PAORi | Kp4874-E | EC600 |
| Imipenem | >32(R) | 32(R) | 8(R) | 1(S) | 0.5(S) |
| Meropenem | >32(R) | 8(R) | 1(S) | 0.125(S) | 0.015(S) |
| Cefotaxime | >128(R) | 128(R) | 8(R) | 8(R) | 0.125(S) |
| Cefepime | >128(R) | 128(R) | 0.5(S) | 4(I) | 0.03(R) |
| Ceftazidime | >128(R) | >128(R) | 1(S) | >128(R) | 0.5(S) |
| Piperacillin/Tazobactam | >128(R) | 4(S) | 2(S) | 4(S) | 4(S) |
| Amoxicillin/Clavulanate | >128(R) | >128(R) | >128(R) | 32(R) | 16(R) |
| Ceftazidime/Avibactam | >128(R) | >128(R) | 4(S) | 128(R) | 0.25(S) |
| Ceftriaxone | >128(R) | 64(R) | 4(R) | 4(R) | 0.06(S) |
| Aztreonam | >128(R) | 16(R) | 1(S) | 0.25(S) | 0.06(S) |
| Ciprofloxacin | >64(R) | >64(R) | 0.5(I) | 0.25(S) | ≤0.04(S) |
| Chloromycin | >128(R) | >128(R) | >128(R) | 8(S) | 8(S) |
| Gentamicin | >128(R) | 4(S) | 1(S) | 1(S) | 0.5(S) |
| Amikacin | >128(R) | 16(S) | 1(S) | 2(S) | 2(S) |
| Levofloxacin | 64(R) | 0.25(S) | 1(I) | 0.25(S) | 0.25(S) |
| Methylene/ Sulfameroxazole | 8(R) | 4(R) | 4(R) | ≤0.125(S) | ≤0.125(S) |
| Tigecycline | 0.5(S) | 1(I) | 1(I) | 0.06(S) | 0.125(S) |
| Polymyxin B | 0.5(I) | 8(R) | 2(I) | 0.06(I) | 0.25(I) |

**Note:** R, resistant; S, susceptible; I, intermediate.
