# Supplementary material for: Emergence of ST11 Klebsiella pneumoniae co-carrying blaKPC-2 and blaIMP-8 on conjugative plasmids
Source: Microbiol Spectr. 2025 Oct 8;13(11):e03345-24. doi: 10.1128/spectrum.03345-24 (PMC12584672; doi:10.1128/spectrum.03345-24)
Supplement: Table S2 — The genomic characteristics of Kp4874. [file spectrum.03345-24-s0005.docx]

Table S2 The Genomic Characteristics of Kp4874

| **Kp4874** | **Sizes** | **Type** | **G+C%** | **ARGs^*^** |
| --- | --- | --- | --- | --- |
| Chromosome | 5,434,439 bp | ST11 | 57.3% | *aadA2b*, *bla*_SHV-182_, *bla*_SHV-159_, *bla*_SHV-158_, *fosA6*, *sul1* |
| p1-Kp4874 | 190,082bp | IncHl1B/ repB | 49.8% | - |
| pKp4874_KPC | 134,873 bp | IncFII/ IncR | 53.3% | *rmtB*, *bla*_KPC-2_, *bla*_CTX-M-65_, *bla*_SHV-12_, *bla*_TEM-1B_ |
| pKp4874_LAP | 86,122bp | Unclassified | 54% | *bla*_LAP-2_, *catA2*, *qnrS1*, *sul2*, *tet(A)*, *dfrA14* |
| pKp4874_IMP | 75,644bp | Unclassified | 55.2% | *aac(6')-Ib-Hangzhou*, *bla*_IMP-8_ |
| P6-Kp4874 | 42,152bp | ColRNAl | 58% | - |
| P7-Kp4874 | 11,970bp | Unclassified | 55.6% | - |
| P8-Kp4874 | 6,796bp | Unclassified | 51.6% | - |

**^*^**ARGs: antimicrobial resistance genes.
