# Supplementary material for: Emergence of ST11 Klebsiella pneumoniae co-carrying blaKPC-2 and blaIMP-8 on conjugative plasmids
Source: Microbiol Spectr. 2025 Oct 8;13(11):e03345-24. doi: 10.1128/spectrum.03345-24 (PMC12584672; doi:10.1128/spectrum.03345-24)
Supplement: Table S3 — Amplification primers for IS26 and TnAs1. [file spectrum.03345-24-s0006.docx]

**Table S3 Amplification primers for IS*26* and Tn*As1***

| Primers name | Primers sequence |
| --- | --- |
| KPC-F  KPC-R  IMP-F  IMP-R | 5’-GTCACTGTATCGCCGTCTAG-3’  5’-GTCACTGTATCGCCGTCTAG-3’  5’-GTAMGTTTCAAGAGTGATGC-3’  5’-GTCACTGTATCGCCGTCTAG-3’ |
| IS*26*-F | 5’-ACTCCACGATTTACCGCTGG-3’ |
| IS*26*-R | 5’-CTGCTTACCAGGCGCATTTC-3’ |
| Tn*As1*-F | 5’-GCGCATCAAGATCACCGAAC-3’ |
| Tn*As1*-R | 5’-GTGTAGTGCTCCTCGATCCG-3’ |
