# Supplementary material for: Emergence of ST11 Klebsiella pneumoniae co-carrying blaKPC-2 and blaIMP-8 on conjugative plasmids
Source: Microbiol Spectr. 2025 Oct 8;13(11):e03345-24. doi: 10.1128/spectrum.03345-24 (PMC12584672; doi:10.1128/spectrum.03345-24)
Supplement: Table S4 — Integron Finder analysis results. [file spectrum.03345-24-s0007.docx]

**Table S4 Integron Finder analysis results**

| # integron_finder 2.0.5 | | | | | | | | | |
| --- | --- | --- | --- | --- | --- | --- | --- | --- | --- |
| # cmd: integron_finder L4874hy.fasta --local-max --func-annot --cpu 96 | | | | | | | | | |
| ID_integron | ID_replicon | pos_beg | pos_end | strand | evalue | type_elt | annotation | model | type |
| 01 | 1 | 2767513 | 2767860 | -1 | 2.1999999999999997e-62 | protein | SMR_qac_E-NCBIFAM | NF000276.2 | complete |
| 01 | 1 | 2767963 | 2768022 | -1 | 1.7e-07 | attC | attC | attc_4 | complete |
| 01 | 1 | 2768024 | 2768803 | -1 | 3.2e-152 | protein | ANT_3pp_I-NCBIFAM | NF012157.0 | complete |
| 01 | 1 | 2768961 | 2769851 | 1 | 3.8e-23 | protein | intI | intersection_tyr_intI | complete |
| 01 | 4 | 57079 | 57783 | -1 | NA | protein | protein | NA | complete |
| 01 | 4 | 57950 | 58035 | -1 | 0.064 | attC | attC | attc_4 | complete |
| 01 | 4 | 58030 | 58503 | -1 | 3.4e-93 | protein | trim_DfrA1_like-NCBIFAM | NF000330.1 | complete |
| 01 | 4 | 58659 | 59759 | 1 | 1.0999999999999998e-24 | protein | intI | intersection_tyr_intI | complete |
| 01 | 5 | 15775 | 16476 | -1 | 4.6e-132 | protein | AAC_6p_Ib-NCBIFAM | NF033074.0 | complete |
| 01 | 5 | 16348 | 16426 | -1 | 0.0028 | attC | attC | attc_4 | complete |
| 01 | 5 | 16936 | 18285 | 1 | NA | protein | protein | NA | complete |
| 01 | 5 | 18375 | 19052 | -1 | 6e-153 | protein | blaIMP-NCBIFAM | NF012147.1 | complete |
| 01 | 5 | 19268 | 20281 | 1 | 8.1e-25 | protein | intI | intersection_tyr_intI | complete |
